# Impact of regulatory perturbations to disease spread through cattle movements in Great Britain

**DOI:** 10.1016/j.prevetmed.2011.12.016

**Published:** 2012-06-01

**Authors:** Matthew C. Vernon, Matt J. Keeling

**Affiliations:** School of Life Sciences, University of Warwick, Gibbet Hill Road, Coventry, United Kingdom

**Keywords:** Infectious disease epidemiology, Disease control policy, Simulation

## Abstract

During the past decade the British livestock industry has suffered from several major pathogen outbreaks, and a variety of regulatory and disease control measures have been applied to the movement of livestock with the express aim of mitigating the spread of infection. The Rapid Analysis and Detection of Animal-related Risks (RADAR) project, which has been collecting data on the movement of cattle since 1998, provides a relatively comprehensive record of how these policies have influenced the movement of cattle between animal holdings, markets, and slaughterhouses in Britain. Many previous studies have focused on the properties of the network that can be derived from these movements – treating farms as nodes and movements as directed (and potentially weighted) edges in the network. However, of far greater importance is how these policy changes have influenced the potential spread of infectious diseases. Here we use a stochastic fully individual-based model of cattle in Britain to assess how the epidemic potential has varied from 2000 to 2009 as the pattern of movements has changed in response to legislation and market forces. Our simulations show that the majority of policy changes lead to significant decreases in the epidemic potential (measured in multiple ways), but that this potential then increases through time as cattle farmers modify their behaviour in response. Our results suggest that the cattle industry is likely to experience boom-bust dynamics, with the actions that farmers take during epidemic-free periods to maximise their profitability likely to increase the potential for large-scale epidemics to occur.

## 1 Introduction

The movement of animals within the United Kingdom (UK) is vital to the economics of the livestock industry, but carries with it the risk of transmitting infectious diseases across substantial geographic distances (Haydon et al., 2003; Gilbert et al., 2005; Kiss et al., 2006; Green et al., 2006, 2008; Carrique-Mas et al., 2008). For this reason, data on the movement of all cattle in the UK are collected by the Department for Environment Food and Rural Affairs (DEFRA), as part of the RADAR system, itself part of the Veterinary Surveillance Strategy (Lysons et al., 2007) (strictly, data are collected on the movement of all bovines including water buffalo, bison, and brahman, however throughout we will use the term cattle due to its greater familiarity). These movement data may then be abstracted into different forms of network. The most straightforward is a directed contact network in which agricultural premises are nodes, and the movements of cattle between premises form edges (Kiss et al., 2006; Robinson et al., 2007a). These networks may then be analysed using a range of techniques, including those developed for handling social networks (Wasserman and Faust, 1994; Carrington et al., 2005). Often, to ease computation, the network is considered as static – using all movements over a given period to generate an invariant network (Kiss et al., 2006; Robinson et al., 2007a; Green et al., 2008). However a network is most naturally considered as dynamic (reflecting the fact that links between farms are only present when animals move) and weighted (reflecting the impact of moving different numbers of animals) (Vernon and Keeling, 2009). In fact, the predictions from a more realistic weighted-dynamic network may substantially deviate from predictions made by the simpler static models (Vernon and Keeling, 2009). However, all network approaches share a common assumption: that each farm can be treated as a single node in the network with identical infection dynamics within each node – often assuming that a fixed proportion of animals on each farm are infected.

An alternative approach that recognises the individual nature of livestock is to generate a dynamic network that links individual cattle that currently occupy the same location (farm) with the weight of a link scaled to produce frequency-dependent transmission. In network terminology we have defined a bipartite graph, with edges connecting cattle to the farm that they occupy on a particular day. This leads to a fully individual-based stochastic model of infection transmission within farms that includes the movement of cattle to transfer infection between farms (Keeling et al., 2010). As such, this model is far more attuned to both heterogeneities in the number of cattle per farm and the possibility of within-farm epidemics. Here we use this individual-based approach to evaluate the potential epidemiological impact of changes in the pattern of cattle movements over the past decade. To achieve this, we calculate the mean and distribution of epidemic sizes in each year from 2000 to 2009 inclusive. In this manner, rather than merely modelling an infectious disease process, we are effectively using our large-scale simulation model as a statistical tool to evaluate the impact of movement control regulations.

Movements of cattle have not occurred in an unchanging regulatory environment. In particular, there have been three infectious disease outbreaks (foot-and-mouth in 2001 (Gibbens et al., 2001; Keeling et al., 2001) and 2007 (Cottam et al., 2008), and bluetongue in 2007 (Gloster et al., 2008)) that have necessitated legislation regarding the movement of livestock (Gibbens et al., 2001; Cottam et al., 2008; Hateley, 2009). In addition, other rules concerning the movement of cattle that either strengthen or relax controls have also been introduced during this period, such as those in response to the increasing prevalence of bovine tuberculosis. Each of these has had a complex effect on the pattern of cattle movements that emerges as livestock farmers react to the new rules. Table 1 summarises the main legislation affecting the cattle industry in Britain since 2001; the changes in movement patterns themselves in the last decade have been considered in detail by Vernon (2011).

## 2 Materials and methods

### 2.1 Simulation model

Movement data of individual cattle for the period 01/01/2000 to 31/12/2009 were considered. The simulation model deployed for this work is a stochastic discrete-time SEIR model, formulated at the level of individual cattle (Keeling et al., 2010). This model operates on a daily time-scale which is ideal given the daily pattern of activity on livestock farms and that the timing of most movements is only known at the resolution of a particular day. The underlying dynamics of the model are best conceptualised by considering the stochastic processes influencing a given individual animal. For each animal *a* there are three state variables, its location *x*_*a*_(*d*) (which is generally the farm number and depends on the current day, *d*), its status *σ*_*a*_(*d*) (which can be either susceptible, infected or recovered) and, if applicable, the day on which the animal was infected *D*_*a*_. The location parameters *x*_*a*_(*d*) are fixed by the recorded pattern of movements, while the other two parameters are stochastic variables that are derived by simulation.

In particular, the force of infection (*λ*_*a*_(*d*)) for a susceptible animal *a*, on day *d*, is given by the summing over the transmission rates of all infected animals that are located on the same farm at the same time and are currently infectious, and dividing by the number of animals on that farm at that time. The transmission rate from each infected animal varies as a function of the time since the animal was infected, and is therefore able to capture a range of plausible epidemiological behaviours. However, in this paper we assume fixed-duration latent (exposed) and infectious periods (by default of five days each) and a fixed transmission rate (by default 1) during the infectious period; thus generating a within-farm basic reproductive ratio of *R*_0_ = 5. The default choice of durations and transmission rates was chosen such that there was a substantial risk of onward transmission from an index farm, yet the overwhelming majority of epidemics complete within a single year.

All simulations begin on the first of January for the particular year, and are halted when the disease dies out or at the end of December, whichever is earlier. We record which animals and farms have infection at every point in the simulation. Two different seeding scenarios were considered. Firstly a random farm with more than five cattle was picked at the start of the year and five randomly selected cattle were infected. For many initial seeds the infection rapidly dies out; this initial condition therefore provides a measure of invasion potential and early epidemic growth rate for a particular year. In a second formulation, one thousand randomly selected cattle were initially infected; this provides some measure of the ability of the movement network to support a more widespread infection.

### 2.2 Network measures

In network theory, the giant component size refers to the largest number of nodes (farms) that are inter-connected by network links—and has frequently been used to assess the epidemiological potential of a network (Robinson et al., 2007a; Kao et al., 2006a; Rautureau et al., 2011). There are two main ways in which these inter-connections can be considered, referred to as giant weak and giant strong components. For the type of directed network considered here (where connections have a definite direction associated with them), the giant strong component is the largest set of farms where there is a forward path from every farm to every other farm – in principle infection due to movement can spread from any farm to any other farm (possibly via intermediate farms) in the component. The giant weak component is similarly defined except the direction of the contact between farms is ignored. These concepts may be more formally defined in terms of the graph of network connections (Wasserman and Faust, 1994).

Obviously the size of giant components can be calculated from any network; in particular, different length “observation windows” can be used to generate static networks from all the movements during that period. The sizes of giant components using different observation windows of UK cattle movements have been previous used as proxy measures for the risk of disease spread within the UK cattle herd (Robinson et al., 2007a). Here we calculate the size of giant weak and strong components for a variety of observation windows: a 10-day window (the sum of the latent and infectious periods, as suggested by Kao et al., 2006a); a 28-day window (the same snapshot length as used by Robinson et al., 2007a); and from year-long static networks. Giant component sizes are calculated by using an improved version of Tarjan's algorithm (Nuutila and Soisalon-Soininen, 1993), as implemented in the Contagion software package (Vernon, 2007).

## 3 Results

**Fig. 1:**
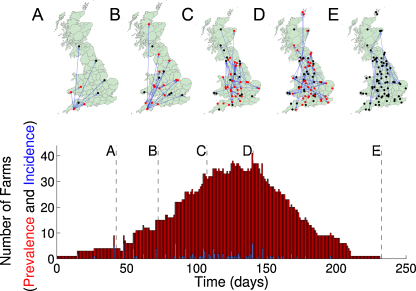
Example epidemic for the movement pattern of 2009, showing the state of farms (red, those containing infected cattle; black, those that previously contained infected cattle) at five time points during the epidemic. Blue lines join newly infected farms to their source of infection. The bottom graph shows the time-course of the epidemic, in terms of the number of farms with infected cattle.

Fig. 1 shows the results of a typical epidemic (begun with five infected cattle on a single farm) that spreads nation-wide. The maps for 5 different time points show the distribution of farms containing infected cattle (red) and ones that have been previously infected (black) together with the movements of cattle that could have transported infection. The lower graph shows the prevalence (number of farms currently infected, red) and incidence (number of farms newly infected, blue) for each day of the epidemic.

### 3.1 Annual patterns and regulations

**Fig. 2:**
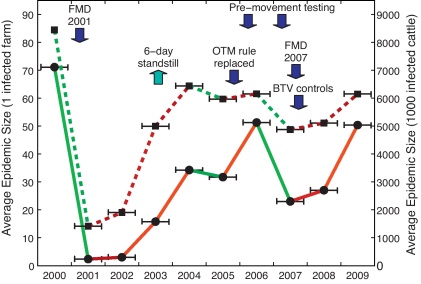
Mean number of cattle infected by epidemics seeded on five cattle on a single farm (solid line, left-hand axis), or on one thousand cattle (dashed line, right-hand axis), based on movement data from 2000 to 2009. Mean values are joined with lines that are colour-coded to reflect the magnitude of the change. Results are generated from over 23,000 individual-level simulations for each year, and hence the confidence intervals on the means are very small. Significant changes to movement legislation are illustrated with blue arrows; Table 1 describes these changes in detail.

Fig. 2 shows the results of multiple simulations based on the two different seeding scenarios: the solid line shows the results of simulations where just five cattle on a random seed farm were infected; the dashed line shows the results of simulations where one thousand randomly selected individual cattle were infected. Clearly, there is substantial year-by-year variation, with no single temporal trend immediately visible in the data (in contrast to the behaviour postulated by Robinson et al., 2007a); however by highlighting when regulatory changes occurred (blue arrows) we observe that the introduction of new regulations impacts both epidemic measures. A smaller number of simulations were performed using different parameter values; the outcome of this sensitivity analysis is described in the Supplementary data.

### 3.2 Giant components and epidemic sizes

**Fig. 3:**
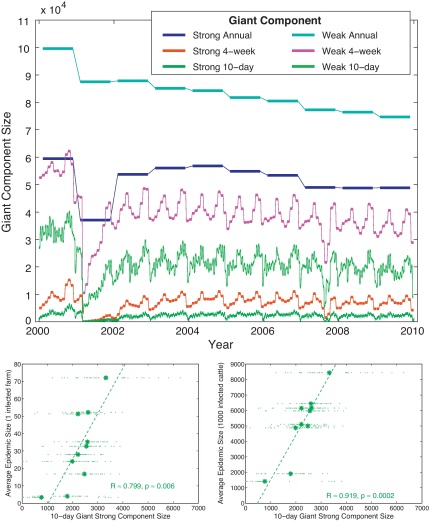
Giant component sizes and their relations to predicted epidemic sizes. Graph a; the size of the giant weak and strong components of networks constructed from consecutive 10-day, 28-day, and year-long snapshots of cattle movements; clearly longer snapshots that contain more movements lead to larger giant components. Graphs *b* and *c*; correlation between mean giant strong component size (averaged over each year) based on 10-day snapshots of movements (shown in dark green in graph *a*) and mean final epidemic size for the two different seeding scenarios of the full simulation. The correlation is highly significant and has the lowest *p*-values for all the snapshots tested (see Supplementary data).

Fig. 3a shows the sizes of the giant weak and strong components based on 10-day, 4-week, and year-long snapshots of movement data. Component sizes from observation windows shorter than one year all clearly show the strong seasonality that is present in the cattle movement network, and which must impact on the success of any disease invasion. The correlation between the mean giant component size (averaged over each year, for a variety of observation windows) and the mean predicted epidemic size is considered in the Supplementary data. Of all the component sizes considered, only the average size of the giant strong component calculated from 10-day observation windows shows a highly statistically significant correlation with the mean predicted epidemic size (for both initial conditions: *p* = 0.006 with 5 infected cattle and *p* = 0.0002 with 1000 infected cattle), explaining 63% and 84% of the between-year variation for epidemics begun with 5 and 1000 infected cattle respectively (Fig. 3 b and c).

### 3.3 Future dynamics

**Fig. 4:**
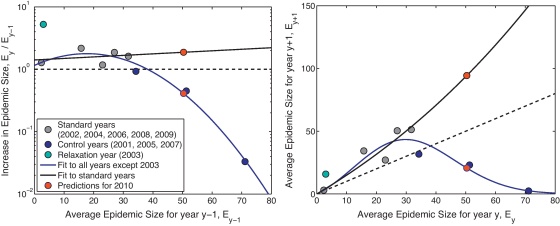
Change in epidemic size between one year and the next. The dots represent average epidemic sizes from multiple stochastic simulations for each year starting with 5 cattle infected on a randomly chosen farm (as in the solid lines in Fig. 2); colours represent the regulatory changes in the year: grey is no change, blue is control implemented, cyan is control relaxed. Black and blue lines represent the best fit polynomial curves (as specified by the Akaike information criterion (Akaike, 1974)) to the relative change in epidemic sizes; the black line is a quadratic fit to the grey points, the blue line is a cubic fit to both the blue and grey points. Finally, the red dots show the predictions for 2010 based on the two best fit curves. (A) The relative change in average epidemic size (plotting *E*_*y*_/*E*_*y*−1_ against *E*_*y*−1_). (B) The relationship between the average epidemic sizes in two consecutive years (plotting *E*_*y*_ against *E*_*y*−1_).

The results of the full simulation model show the tendency for new movement patterns to emerge that alleviate the impact of restrictions; this can be quantified by comparing the size of epidemics in years without new controls (*E*_*y*_) to the size of the epidemic in the previous year (*E*_*y*−1_). This comparison is shown by grey dots (simulation data; results from years with no regulatory change) and black line (best-fit predictor) in Fig. 4, where *E*_*y*_ is taken to be the average size of epidemics in year *y* when one farm (and five cattle) is initially infected. The implication is that in the absence of any new regulatory measures the size of an expected outbreak (should it occur) increases year-by-year (black lines in Fig. 4). However, while it is clear that the movement of cattle, and therefore the spread of infection, is affected by the regulatory environment, there is also a more complex feedback with the spread of infection influencing the need for new legislation (e.g. that large-scale disease outbreaks tend to result in newly restrictive legislation being passed). It is possible, therefore, to consider infection, cattle movement and legislation as a closed system (rather than simply considering years in which regulations have not changed, instead consider infection, movement, and legislation as all influencing each other); in this case predictive models can include data from all years (including those in which epidemics occurred and changes in the regulations were required, shown in blue in Fig. 4). By including these additional points we incorporate the risk of a new epidemic and the potential need for new regulations (as captured by the difference between blue and black lines), and can readily observe how movement patterns that permit large epidemics are unstable.

## 4 Discussion

In this paper we have utilised a highly computationally intensive individual cattle-based simulation model for predicting the stochastic dynamics of infections in the bovine population of Great Britain. The model captures the movements and epidemiological dynamics of all bovine animals from January 2000 until December 2009: a total of over 71 million movements and over 30 million animals. This simulation model is fundamentally simple, tracking the infectious status of every animal and allowing frequency-dependent transmission between all bovine animals that are located on the same farm at the same time. The computational complexity comes from the vast number of animals that must be considered. Given these computational demands (and the need for replicate simulations due to the stochasticity of the system) we have considered only a small set of epidemiological parameters, concentrating most of our computational effort on a single parameter set that corresponds to a relatively fast infection that has a within-farm basic reproductive ratio of *R*_0_ = 5. Reassuringly, the broad pattern of changes between years is consistent across the range of parameters we considered (see Supplementary data for details).

We initially use this model to address a long-standing and important issue in livestock epidemiology (Robinson et al., 2007a): is the cattle industry becoming more susceptible to epidemics over time? We used mean final epidemic size as a measure of susceptibility, and found that in 3 years (2001, 2005 and 2007) there is a decline in both epidemic measures relative to the previous year; in all other years the epidemic measures increase (Fig. 2). These three declining years are all associated with substantial changes in the legislation regarding the movement of livestock. In 2001, the livestock industry in Great Britain was devastated by a nationwide foot-and-mouth epidemic that prevented the overwhelming majority of cattle movements from March to October (Gibbens et al., 2001; Keeling et al., 2001). The decline in 2005 is minimal, and may be associated with the replacement of the over 30 months (OTM) rule that began in 1996 to control the spread of BSE (Ferguson and Donnelly, 2003; Arnold and Wilesmith, 2003); relaxing this rule (which provided compensation for slaughtering cattle that reached 30 months of age) will mean that farmers are more likely to keep older cattle and hence there will be a reduction in movements. Finally, in 2007 there were several issues which impacted on cattle movements: the second phase of pre-movement testing for tuberculosis (Green et al., 2008), the small-scale outbreak of foot-and-mouth disease in Surrey (Cottam et al., 2008), and the spread of bluetongue (Gubbins et al., 2008; Gloster et al., 2008). Not all legislation has a negative impact on epidemic sizes however. In 2003 a 6-day standstill was introduced preventing the movement of cattle off a farm that had moved animals on within the past 6 days; this replaced a 20-day standstill that had been in place since 2001 and was therefore a relaxing of the legislation. In response to this we observe (as anticipated) a sharp rise in both epidemic measures in 2003.

Changes in legislation surrounding the movement of animals (often driven by epidemiological factors) have a pronounced impact on the potential for an infection to percolate through the cattle movement network. This is as expected (and indeed hoped for); regulatory changes that in some way restrict, but still allow the majority of movements, are predicted to limit the spread of infection. What are potentially more interesting are the dynamics in-between these times of regulatory change. Although these new regulations may be long-term or even permanent (such as the pre-movement testing for bTB or the replacement of the over-thirty-month rule), their impact is temporary and waning. From 2000 to 2009, we therefore observe gradually increasing susceptibility of the cattle industry punctuated by new laws which restrict movements and hence lower susceptibility. By taking a longer-term view of the temporal trends in susceptibility, it can be seen that the worryingly large increase in potential epidemic sizes observed when only considering 2002–2005 (Robinson et al., 2007b) was somewhat misleading. We postulate that the increase in susceptibility is due to farmers finding new ways of moving livestock that increase their profits while still conforming to the new regulations; indeed there will be complex economic drivers underlying farmers’ behaviour, which warrant further study. There has been little other work modelling the impact of movement regulations on infectious disease; a Dutch study compared the cattle movement network of the Netherlands in 2000 and 2002 (before and after FMD), and showed that, in the short term at least, regulations had lead to a reduction in the number of live animals moved, and a corresponding reduction in the predicted size of FMD epidemics (Velthuis and Mourits, 2007). It would be valuable to examine how long this protective effect persisted. Further, while there appear to be similar movement patterns within several EU member states (Vernon, 2011), a more systematic comparison of the movement networks of different countries, and how those movement networks relate to the spread of simulated epidemics would provide significant insights for policy making. In particular, it would be valuable to establish whether the problem of regulatory change only resulting in short-term reductions in disease susceptibility is a general one (as would seem likely), or if it is peculiar to the UK.

One major surprise is the fact that the average size of the 10-day giant strong component (an easily calculated and fairly abstract network quantity) can be used as a proxy measure for the predicted epidemic size on an animal movement network (Fig. 3). This is an interesting feature of the structures and patterns within the cattle movement network; it is relatively straightforward to construct simple movement networks where this relationship does not hold. In particular, although there is considerable heterogeneity between the behaviour of individual farms, it must be presumed that this degree of heterogeneity remains largely invariant over the period 2000–2009 such that its impact on the epidemic remains constant; this finding supports the suggestion by Kao and colleagues that epidemic systems on livestock movement networks may be ergodic (Kao et al., 2006b). The calculation of giant strong component size ignores many factors conventionally considered important in epidemiology, such as: the strength of links between farms (in terms of the number of animals moved); the precise timing of movements on and off farms; the persistence of infection on farms (and how this is affected by the number of animals present); and the fact that farms containing mainly recovered cattle can act as a barrier to transmission through the network. Accordingly, much more theoretical research is needed to assess under what conditions an epidemic driven by complex dynamic heterogeneous movement patterns can be approximated by such simple static measures.

Given the computational efficiency of calculating the giant component, which has a single deterministic value for a given observation window, compared to the demands of modelling multiple individual-based stochastic epidemics, it is justifiable to question our earlier approach. In defence of the stochastic models we raise three main points. The first is that without the modelling results we would have no *a priori* reason to assume that the average giant strong component would necessarily relate to the expected epidemic size; in particular it is not clear that this relationship holds for all epidemiological parameters. As a corollary of this, epidemic modelling results are required if we wish to translate between giant component sizes and the expected number of cases that are likely to be observed. Secondly, the stochastic nature of the epidemic simulations provides far more information than the giant component size; allowing us to predict the distribution of epidemic sizes and also the likely temporal pattern of cases. Finally, the simulations generate information on individual farms, highlighting which farms (or even which type of farms) are most likely to suffer the greatest burden of infection. Such information is vital in all aspects of surveillance and control (Dubé et al., 2009); additionally, it may be used to validate network measures which have been employed to estimate particular nodes’ susceptibility to infection (for a review of some of these, see Danon et al. (2011)). In summary therefore, while the 10-day giant component size provides a simple measure for quantifying epidemiologically important changes in the network structure over relatively short time-scales, it is only through the use of fully individual-based simulations (that account for the full range of heterogeneities and epidemic processes) that reliable predictions can be made.

While it is scientifically interesting to examine existing patterns, it is far more policy-relevant to concentrate on the likely future dynamics. Predictions for the potential epidemic size in 2010 (red dots, Fig. 4) and into the future depend on the underlying premises about disease invasion. If there are many potential invasions each year, which simply do not propagate until the movement network is sufficiently interconnected, then the behaviour is largely predicted by the blue curve and we should expect a significant outbreak and associated movement controls in the near future. In contrast, if invasions are themselves relatively rare, then we may see several years before a new outbreak occurs. Given that 2010 was free of a major livestock epidemic, the latter assumption seems most probable. What is clear, however, is that the current pattern of movements is such that endemic diseases are likely to increase and that any novel infections that do invade are likely to spread.

In summary, given the reactionary way that legislation can only respond once an outbreak has become apparent, and the economic pressure for farmers to freely move cattle, the UK livestock industry is likely to be subject to a repeated cycle of epidemics and new regulatory controls. Devising regulations that can break this cycle is an open challenge for epidemiologists and policy makers, who must in the mean time bear in mind that any new regulatory intervention will have a transient impact.

## 5 Conclusions

Two main conclusions emerge from these simulations. The first is that movement restrictions have a substantial impact on the way an epidemic can percolate through the cattle industry, and are therefore an effective means of either controlling endemic infections or reducing the industry's susceptibility to invading pathogens. The second is that the impact of such measures does not persist for the lifetime of the legislation, but decays through time. The profits made within the cattle industry are fundamentally linked to the movement of animals, and the greatest profit can be made when there is complete freedom to move animals at any time and to any place. This generates strong economic drivers for cattle farmers to develop new movement patterns that alleviate the burden of any restrictions, which in turn lead to a constant tension between profit and disease prevention.

## Conflict of interest statement

The authors declare that they have no conflicts of interest.

## Figures and Tables

**Table 1:** Legislation that has affected livestock movement in the UK since 2001.

Short name	Legislation	Date	Effect
FMD 2001	Various	February 2001 to October 2001	Restrictions on the movement of all livestock for the duration of the epidemic. 20-day standstill enforced on all premises until August 2003, i.e. farms moving livestock on cannot move livestock off for 20 days

6-Day standstill	Disease Control (England) Order 2003	August 2003	Farms moving livestock on cannot move livestock off for 6 days

Over 30 months (OTM) rule	–	November 2005–	OTM rule replaced by testing for BSE and compensation scheme cancelled.

Pre-movement testing	Tuberculosis (England/Wales/Scotland) Order 2006	March 2006–	Cattle on a farm with a 1- or 2-year bTB testing interval being moved must have been tested for bTB within 60 days

	Tuberculosis (England) Order 2007	March 2007–	
FMD 2007	–	August–September 2007	Restrictions on movements due to localised outbreak in Surrey

BTV controls	Various	September 2007–	Following the identification of BlueTongue virus on 21 September, a variety of spatial movement restrictions were enacted
