# Supplementary material for: Impact of regulatory perturbations to disease spread through cattle movements in Great Britain
Source: Prev Vet Med. 2012 Jun 1;105(1-2):110–7. doi: 10.1016/j.prevetmed.2011.12.016 (PMC3343271; doi:10.1016/j.prevetmed.2011.12.016)
Supplement: Supplementary file 1 [file mmc1.pdf]

Impact of Regulatory Perturbations to Disease Spread through Cattle Movements in Great Britain
Supplementary Material

Matthew C. Vernon   Matt J. Keeling

School of Life Sciences, University of Warwick, UK

Simulation Model

Movement data of individual cattle for the period 01/01/2000–31/12/2009 were considered. The simulation model deployed for this work is a stochastic discrete-time SEIR model, formulated at the level of individual cattle. This model operates on a daily time-scale which is ideal given the daily pattern of activity on livestock farms and that the timing of most movements is only known at the resolution of a particular day. The underlying dynamics of the model are best conceptualised by considering the stochastic processes influencing a given individual animal. For each animal a there are three state variables, its location $x_a(d)$ (which is generally the farm number and depends on the current day, d), its status $\sigma_a(d)$ (which can be either susceptible, infected or recovered) and, if applicable, the day on which the animal was infected D_a. The location parameters $x_a(d)$ are fixed by the recorded pattern of movements, while the other two parameters are stochastic variables that are derived by simulation.

In particular, the force of infection ($\lambda_a(d)$) for susceptible animal a, on day d, is given by the sum over all infected animals that are located on the same farm at the same time and are currently infectious:

$$\lambda_a(d) = \frac{\displaystyle\sum_{b, x_b(d)=x_a(d), \sigma_b=I} \beta(d - D_b)}{\displaystyle\sum_{b, x_b(d)=x_a(d)} 1}.$$

Here the numerator captures how the rate of transmission (β) varies as a function of the time since the animal was infected, while the denominator (which is simply the

number of animals on the same farm) ensures frequency-dependent transmission. The probability that a given susceptible animal a is infected on day d is given by:

$$IP(a \text{ infected on day } d) = 1 - \exp(-\lambda_a(d))$$

thereby translating the continuous time rate into a probability. If this probability is realised then the status of the animal is changed to infected ($\sigma_a = I$) and the day of infection is set ($D_a = d$). For simplicity throughout this paper, we assume the transmission rate, β, to be a step function:

$$\beta(D) = \begin{cases} \tau & \text{if } 5 < D \leq 10 \\ 0 & \text{otherwise} \end{cases}$$

this ensures a latent (exposed) period of five days followed by an infectious period of five days, after which time the animal is assumed to possess perfect, lifelong immunity. Except where otherwise specified, $\tau = 1$. This mixture of durations and transmission rates was chosen such that there was a substantial risk of onward transmission from an index farm, yet the overwhelming majority of epidemics complete within a single year.

Sensitivity to Epidemiology

The results given in the main paper are derived for a theoretical pathogen whose behaviour was parameterised to achieve a balance between likely invasion and short-duration epidemics, such that annual values could be generated and therefore annual variation considered. In figure S1 we briefly consider the sensitivity of these finding to the general epidemiological parameters. For a particular pathogen of concern, we would obviously want to chose parameters that accurately reflect the known epidemiology; however parameterising the model for a range of cattle pathogens is infeasible and would not account for farm-level actions taken to control the infection. We therefore limit our analysis to the sensitivity of our previous results to changes in the within-farm basic reproductive ratio (R_0) and changes in the duration of exposed (latent) and infectious periods (figure S1).

Two immediate features from this sensitivity analysis are: firstly the average epidemic size increases with both R_0 and the duration of the periods; and secondly that the general trends of increasing or decreasing epidemic potential from one year to the next remain. In particular, short latent and infectious periods would be expected to lead to relatively short within-farm epidemics and therefore far less chance that an epidemic can spread beyond the initial source. Low values of R_0 tend to be associated with a smaller final epidemic size within a farm (cf work on within-household dynamics for human infections Ross et al. (2010)) which again reduces the potential for between-farm transmission, while a pathogen with a high R_0, and therefore a high transmission rate, is likely to trigger a within-farm epidemic even if it is only present on the farm for a short duration.

Although the sensitivity analysis generally shows consistent patterns in response to movement legislation, there are subtleties that hint at a deeper understanding of the epidemics that occur. We note that the average epidemic size for 2001 is substantially larger when the periods are just 2 days; this is because there is sufficient time for a sizable epidemic to occur between the introduction of the pathogen on 1st January and the imposition of movement restrictions due to the foot-and-mouth outbreak in late February. In addition, low R_0 and longer duration infections tend to spread comparatively better under more recent movement patterns than those at the beginning of the decade. This suggests that such slow infections may have been preferentially selected for by recent movement regulations.

Epidemics or Early Extinction

In any stochastic epidemic, starting with a small number of initially infected cases, the distribution of final epidemic sizes is generally bimodal. In some scenarios the infection is able to spread and a relatively large epidemic is observed, while in others only a few (or even none) additional cases are generated before the epidemic goes extinct. Both of these processes contribute to the average epidemic size that has been reported in the paper. Figure S2 shows the percentage of simulated outbreaks, starting with five infected cattle in a single farm, which infect at least one other farm (blue) or which manage to spread to at least ten other farms (red). Two striking patterns emerge. The first is that 2001 is associated with a high probability of early epidemic spread, this is because this early behaviour occurs in January or February of 2001 before the control measures against Foot-and-Mouth Disease were implemented. The second clear pattern is that the chance of the initial outbreak leading to onwards transmission generally increases through time, although with small fluctuations caused by changes to the movement regulations (again denoted by arrows). What is therefore clear is that while movement regulations have a major impact on the scale of epidemics should they occur, their impact is less pronounced on the chance of infection propagating from the initially infected farm.

Changes in Movement Patterns Over Time

The cattle movement network consists of between 5.2 million and 10.4 million cattle movements per year linking the 361 thousand cattle farms in Great Britain. This produces a dynamic weighted directed network between these farms, which can be analysed with a variety of techniques. We consider a range of these measures below and describe the correlation between the measure and the predictions of the full stochastic model. Given that all these measures treat each node (farm) as identical units, it is intuitively clear that they cannot fully capture the intrinsic heterogeneities in the behaviour

of epidemics within farms of different sizes.

Distance Moved

The cattle movement network is not an abstract concept but instead relates to the real movement of animals between two physical locations. The distance of each move can be calculated, and it might be hypothesised that longer movements allow an infection to percolate through the cattle industry more easily. The distances of movements across the years 2000–2009 are shown in figure S3 (main graph); the mean, median, quartiles and 95th percentiles are plotted. The two sub-graphs show the relationship between the mean distance moved and the mean epidemic size predicted by the model for the two initial conditions discussed in the main paper. Although mean distance moved and mean epidemic size are positively correlated, as expected, this is not statistically significant and strongly relies on the extreme behaviour in 2000.

Batch Size

In general, cattle are not commonly moved individually, but are frequently sold in batches — this increases the instantaneous strength of a network link between two farms as moving two cows is more likely to transfer infection compared to moving a single animal. The change in movement batch sizes across the years 2000–2009 is shown in figure S4. Other than the outlying behaviour in 2000, there has been little change in the number of cattle per batch over time. This is reflected in the statistically weak correlation between mean batch size and mean epidemic size for each year.

In and Out Degree

If we consider the cattle movements as a network, one of the most intuitive measures is to focus on the number of farms that animals are purchased from or sold to, as this corresponds to the number of potential sources of infection or the number of secondary farms that could be infected. In terms of network theory, and considering all the movements from one year as generating a static binary (unweighted) network, for each farm the number of other farms linked by purchases is given by the in and out degrees of that farm. The means, medians, quartiles and 95th percentiles for both measures (across all farms sending or receiving at least one animal) are shown in figure S5. We again find that there is no statistically significant correlation between either of these measures and the predicted epidemic size for each year.

Number of Cattle in and out

Similarly we may wish to consider the number of cattle entering or leaving each premise as this corresponds to the number of potential transmission events onto or off a farm. In terms of network theory, and considering all the movements from one year as generating a weighted static network, the number of cattle movements for each farm is given by the sum of the row and column of this network containing that farm. The means, medians, quartiles and 95th percentiles for both measures are shown in figure S6. Again, no statistically significant pattern is discernible.

Giant Component Sizes

The correlation between the mean giant component size (averaged over each year) and the mean epidemic size is shown in figure S7. The values of the giant components from 2000 to 2009 is given in figure 4 of the main paper. We note that the average size of the giant strong component calculated from 10-day observation windows (highlighted in figure S7) shows a highly statistically significant correlation with the mean predicted epidemic size (for both initial conditions), explaining 63% and 84% of the between-year variation for epidemics begun with 5 and 1000 infected cattle respectively. In contrast, the size of the giant weak and strong components calculated from other observation windows show less significance. These findings are discussed further in the main paper.

References

Ross, J. V., House, T., and Keeling, M. J. (2010). Calculation of disease dynamics in a population of households. *PLoS ONE*, 5(3).

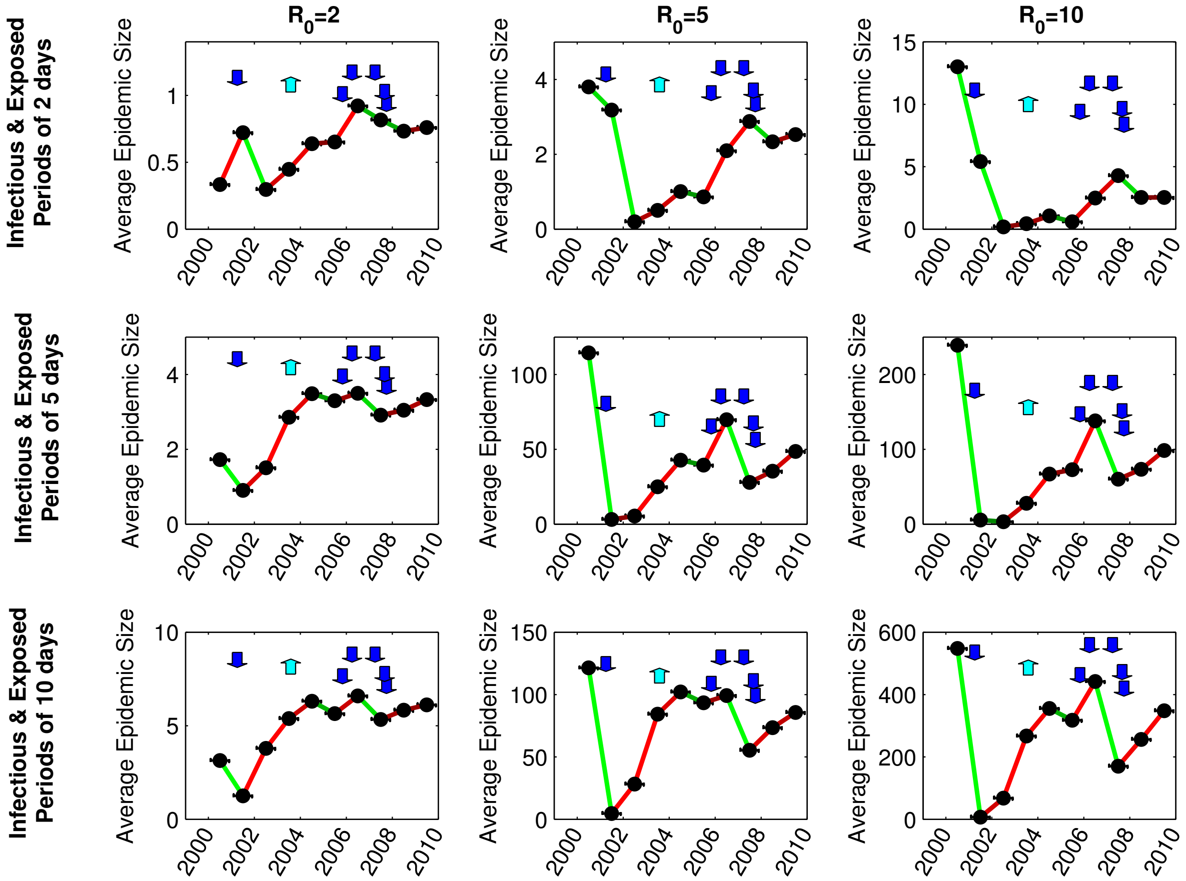

Figure S1: Mean number of cattle infected by epidemics seeded on five cattle on a single farm based on movement data from 2000–2009. Results in each subgraph are comparable with those in figure 2 in the main paper, but explore sensitivity to epidemiological parameters. Columns correspond to different values of within-farm basic reproductive ratios ($R_0 = 2, 5, 10$) while rows correspond to different latent and infectious periods (assumed equal and set to 2, 5 and 10 days). Once again arrows highlight when changes to the movement legislation occurred.

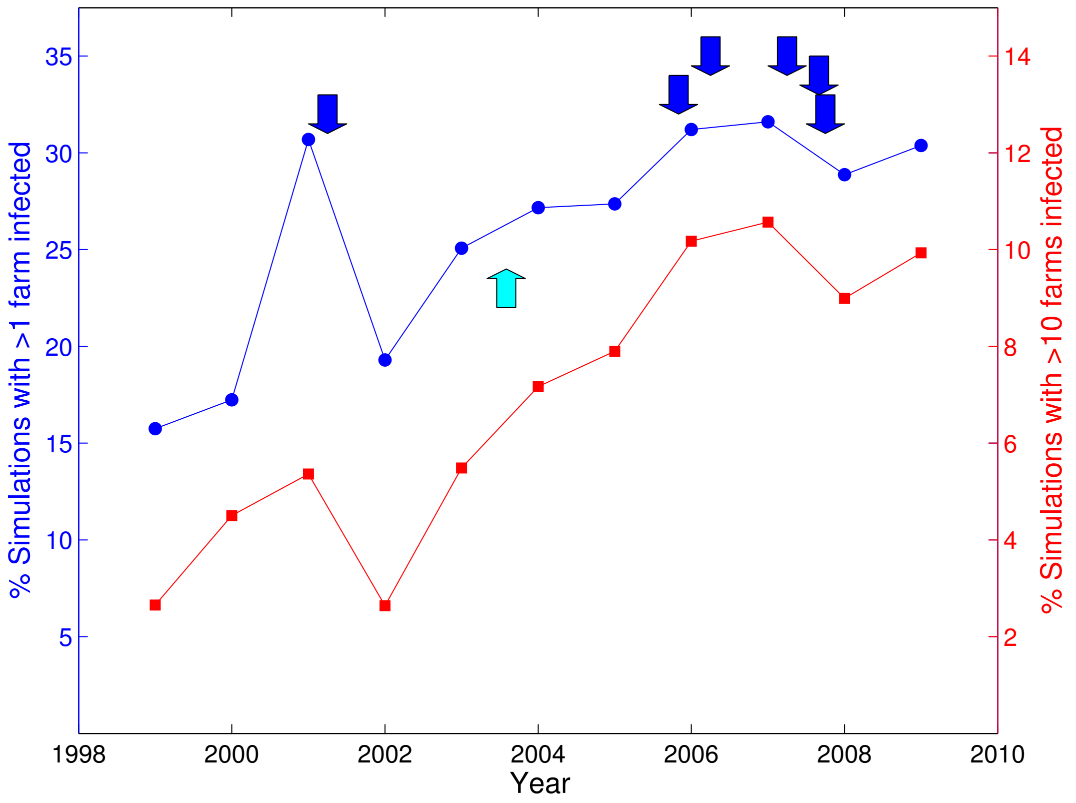

Figure S2: Proportion of epidemics, seeded on one initial farm, that go on to infect at least one (blue line) or at least ten (red line) further farms. Arrows indicate regulatory changes, as described in the main paper.

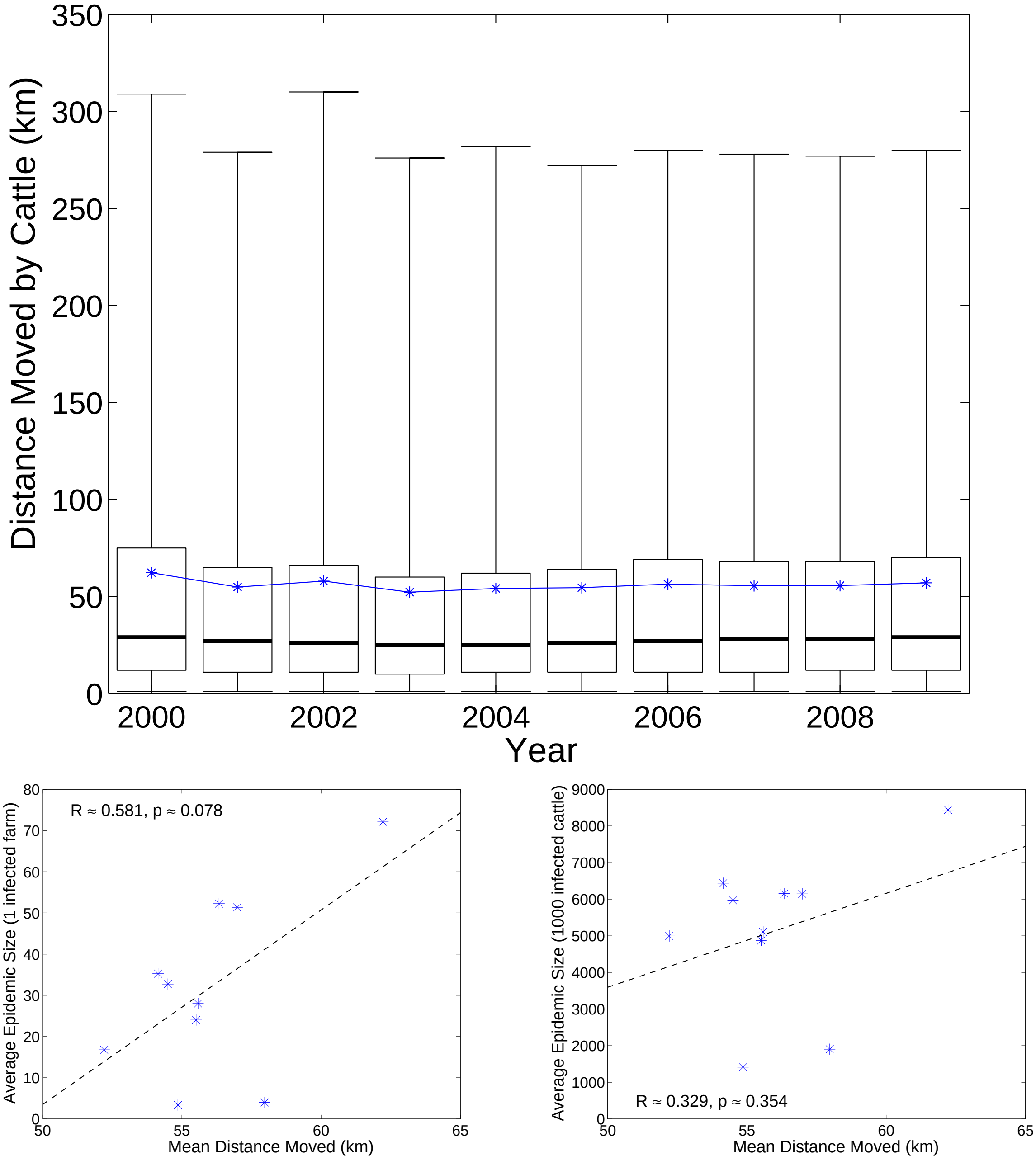

Figure S3: Change in distances animals are moved over time. Box whisker plots show the median, quartiles and ninety-fifth percentiles, the blue line shows the mean value. The bottom plots consider whether mean distance moved is a good predictor of the average epidemic size.

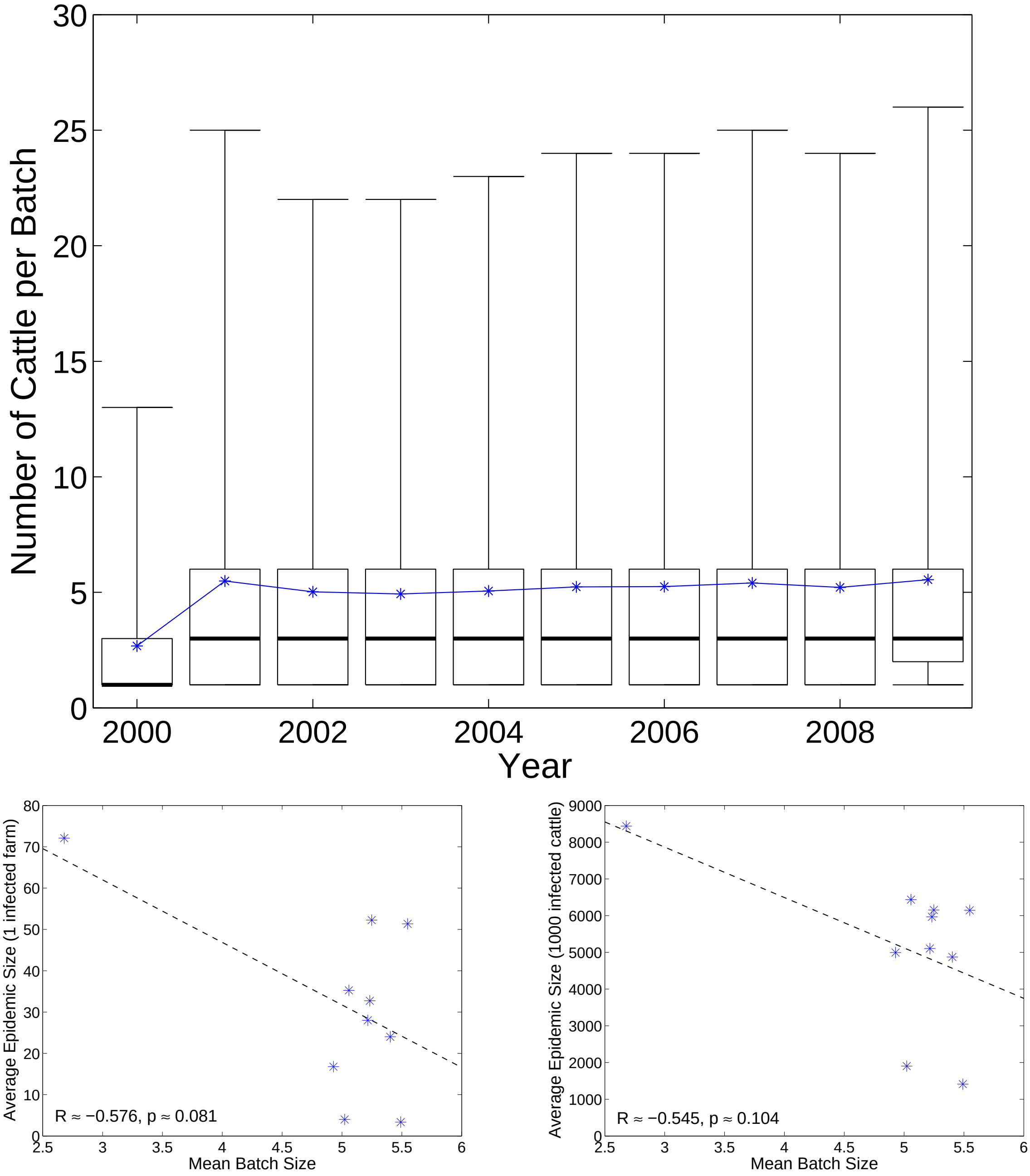

Figure S4: Change in movement batch sizes over time. Box whisker plots show the median, quartiles and ninety-fifth percentiles, the blue line shows the mean value. The bottom plots consider whether mean batch size is a good predictor of the average epidemic size.

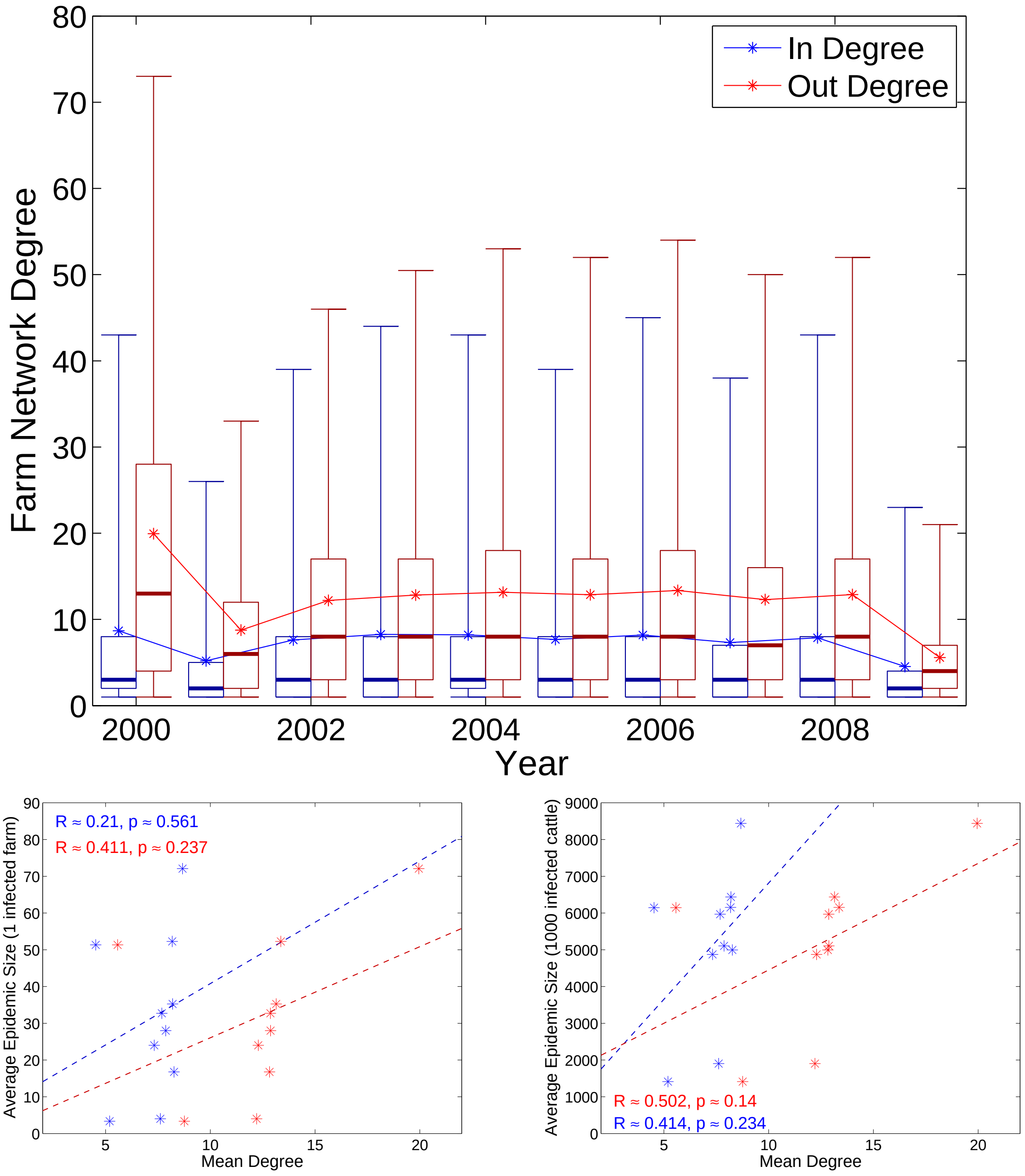

Figure S5: In- and out-degrees of farms per year. Box whisker plots show the median, quartiles and ninety-fifth percentiles, the solid lines show the mean value. The bottom plots consider whether mean in- or out-degree is a good predictor of the average epidemic size.

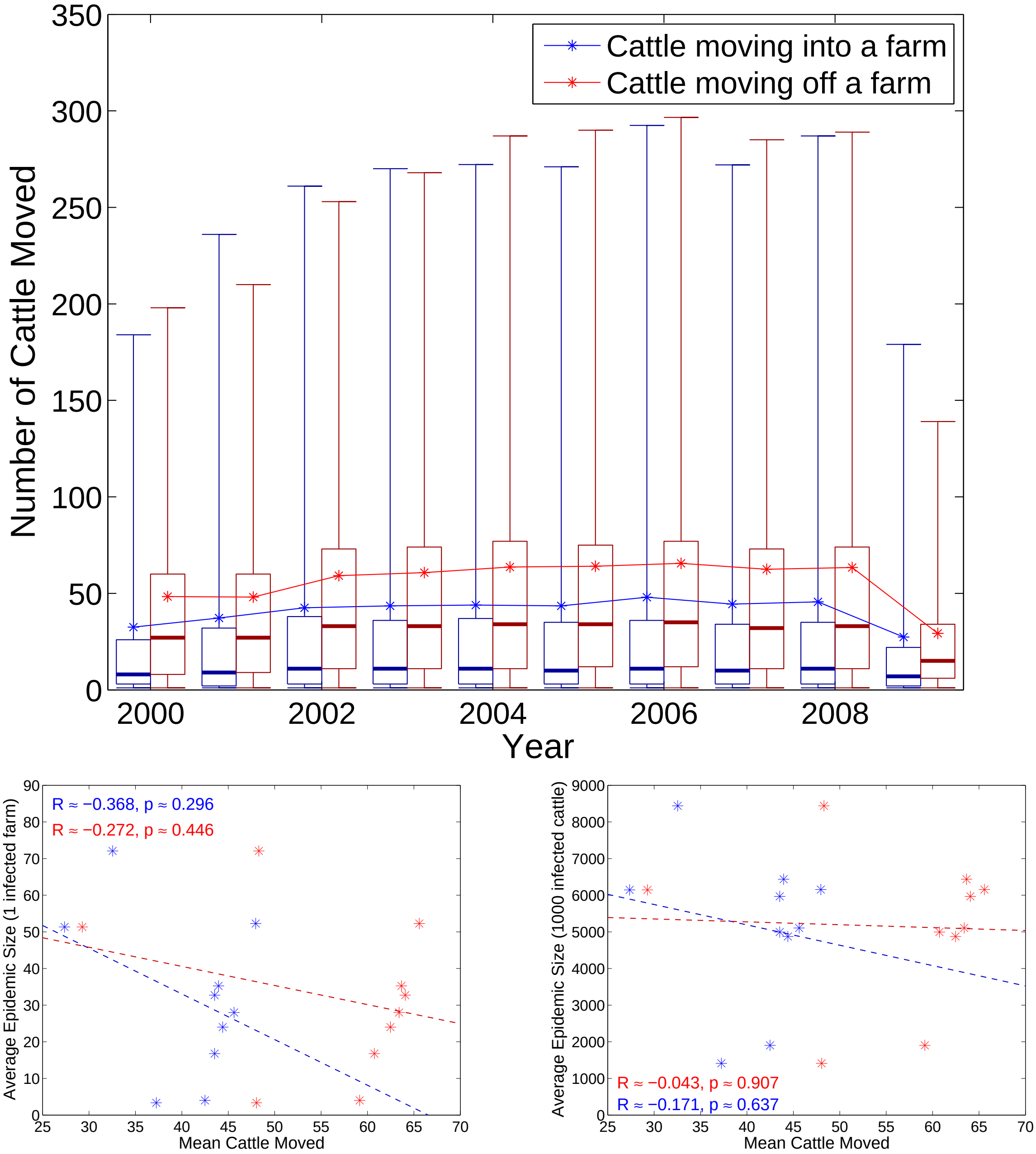

Figure S6: Numbers of animals moved on and off farms per year. Box whisker plots show the median, quartiles and ninety-fifth percentiles, the solid lines show the mean value. The bottom plots consider whether mean number of animals moved is a good predictor of the average epidemic size.

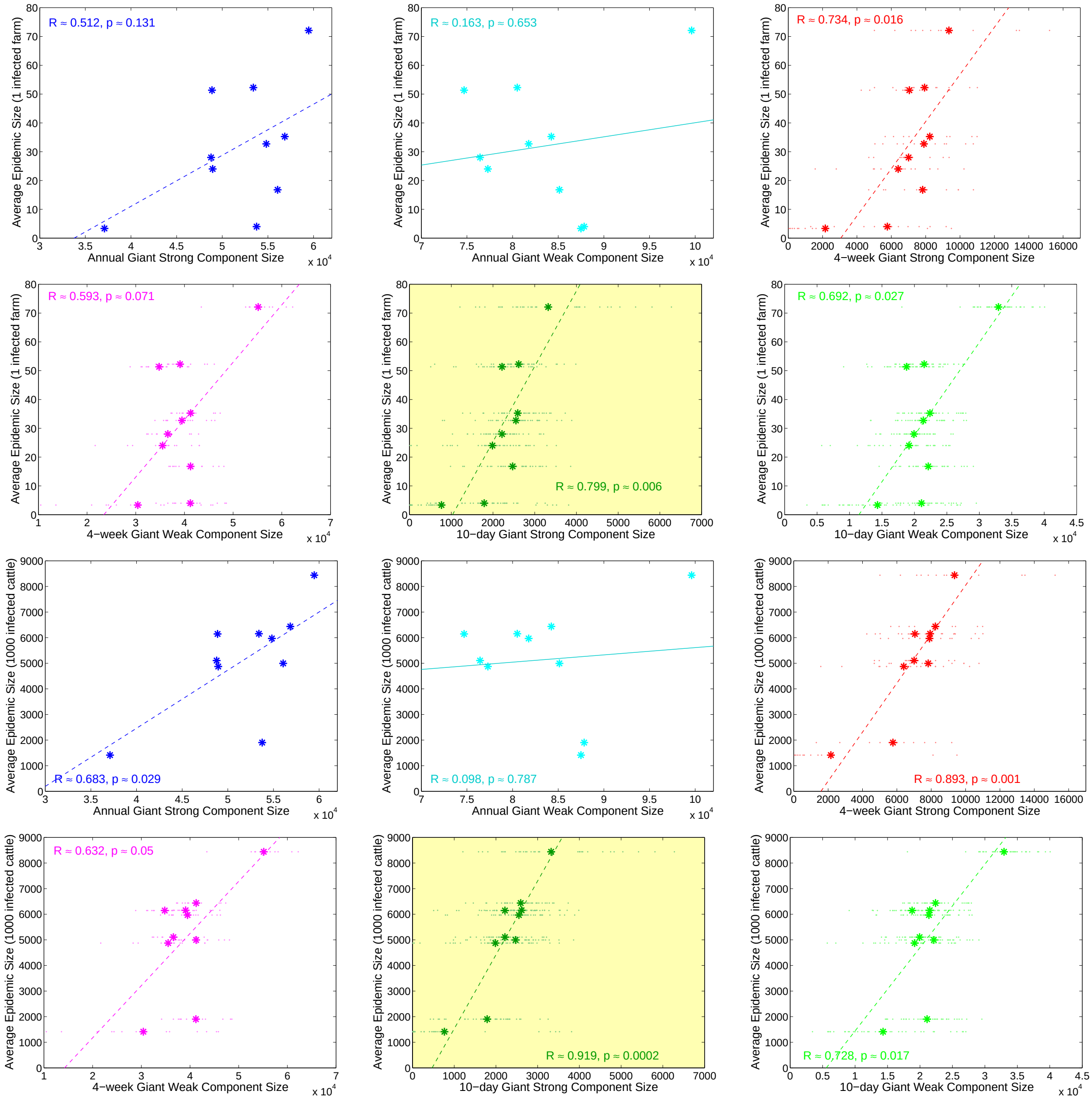

Figure S7: These plots show the degree to which the average giant weak or strong components over a year is a good predictor of the average epidemic size in the two seeding scenarios.
